# Thalassemia: A Review of the Challenges to the Families and Caregivers

**DOI:** 10.7759/cureus.32491

**Published:** 2022-12-13

**Authors:** Rabeya Yousuf, Shaima Akter, Salam M Wasek, Susmita Sinha, Rahnuma Ahmad, Mainul Haque

**Affiliations:** 1 Pathology and Transfusion Medicine, Diagnostic Laboratory Service, Hospital Canselor Tuanku Muhriz, Universiti Kebangsaan Malaysia Medical Centre, Kuala Lumpur, MYS; 2 Internal Medicine, 250 Bed District Sadar Hospital, Chattogram, BGD; 3 Pediatrics, Mohammadpur Upazila Health Complex, Magura, BGD; 4 Physiology, Khulna City Medical College and Hospital, Khulna, BGD; 5 Physiology, Medical College for Women and Hospital, Dhaka, BGD; 6 Pharmacology and Therapeutics, National Defence University of Malaysia, Kuala Lumpur, MYS

**Keywords:** thalassemia, hemoglobin, hereditary hemoglobinopathies, red blood cells, congenital autosomal recessive infirmity, β-thalassemia, homozygotes, progressive severe anemia, financial burden, parental stress

## Abstract

Thalassemias are a group of congenital hemoglobin (Hb) disorders distinguished by dwindling or total curtailment of production of one or more globin chains of hemoglobin tetramers, ensuing in unrestrained destruction of red blood cells (RBC) that causes severe anemia. The severity of the disease often remains immensely variable. Children with thalassemia suffer from the disease's consequences and treatment complications. The disease also causes a negative impact on family members, who suffer mentally, socially, financially, and even physically. In this review, we highlight the challenges experienced by the family and caregivers; for instance, repeated blood transfusion as the dominant origin of tissue casualty, morbidity, and fatal clinical outcomes. Treatment modalities regarding thalassemias were not successful until the inception of bone marrow transplantation and gene therapy.

## Introduction and background

**Figure 1:**
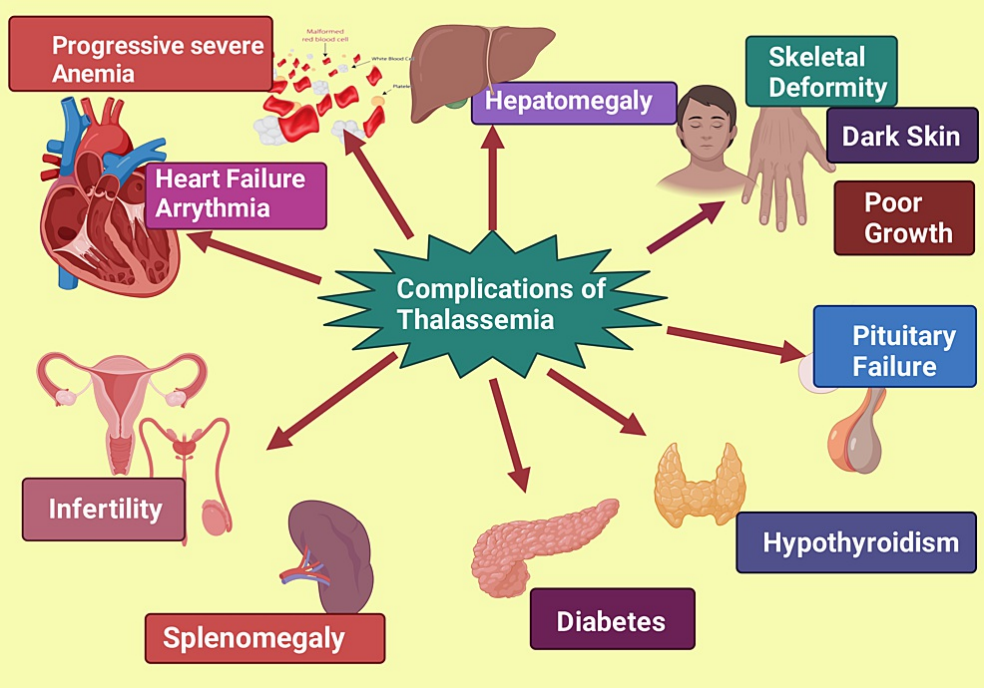
Showing the various complications of thalassemia This figure has been created using the premium version of Biorender (https://biorender.com/) with the publication License no.: MR24QEZXFN. Image Credit: Rahnuma Ahmad.

Thalassemia is derived from the Greek word "Thalassa" meaning sea. This is a congenital autosomal recessive infirmity of hemoglobin (Hb) with a predominant incidence in the Indian subcontinent, Mediterranean and Middle Eastern nations, and Southeast Asia [1]. However, today thalassemia is also prevalent in many countries where it was not recognized before, such as Northern Europe, North Central and South America, and Australia, perhaps due to an improved and fast transport system with immense population migration [2,3]. Thalassemia is an inherited disorder of Hb. There is a reduction or absence of production of one or more globin chains of Hb tetramers, thereby leading to uncontrolled destruction of RBC directed toward grievous anemia [4]. Thalassemias are a diverse cluster of genetic diseases. There are two types of thalassemia, α, and β, which are frequently found. This is based on the involvement of the globin chain [4]. Two versions of the Hb α gene (HBA1 and HBA2) encode an α-chain, and the pair genes are placed on chromosome 16, and Hb β gene encodes the β chain and is located on Chromosome 11 [5]. Another complex form of thalassemia involves either non-functioning or malfunctioning formation of 2-4 non-identical globin sequences [2,3]. Clinically, homozygotes for β-thalassemia often emerge as thalassemia major or intermedia [6]. These patients exhibit progressive severe anemia and extramedullary hematopoiesis leading to poor growth, skeletal deformity, and other complications, including heart failure, hepato-splenomegaly, and regular blood transfusion is the centerpiece of therapeutic intervention for these patients (Figure 1). Homozygous individuals possess two identical semblances of a precise gene, one inherited from each parent; nevertheless, both are abnormal genes [7,8]. β-thalassemia intermedia patients may or may not need blood transfusions in the first two years of life; however, the frequency of transfusions may increase in later life. Thalassemia minor patients present in a carrier state and are usually clinically asymptomatic [9,10].

Screening and antenatal diagnosis showed success in reducing the frequency of new births of β-thalassemia in many Mediterranean countries [11]. Some developed nations have adopted exhaustive national precautionary strategies that embrace mass communication programs to improve cognizance and enlightenment regarding thalassemia. These approaches are supported by carrier identification and advisory program. Additionally, detailed statistics on the advantages of prenatal and preimplantation recognition are also provided [3]. However, accessibility to prenatal screening for diagnosis and access to family planning, as well as denial of therapeutic abortion by the parents due to religious and cultural restrictions, are considered barriers in the prevention program [12].

Epidemiology of hemoglobinopathies

It is reported that globally approximately 300-400 thousand children are born yearly with inherited substantial Hb diseases and around 80 million are carriers of β-thalassemia [2,3,5]. ﻿There are three thalassaemic clinical states with growing disease intensity identified. These are the β-thalassemia carrier state, thalassemia intermedia, and thalassemia major. The β-thalassemia carrier state, which develops because of heterozygosity for β-thalassemia, is clinically symptom-free and with explicit hematological characteristics [13]. Among these global populations, it is estimated that a substantial number of β-thalassemia carrier cases originate from the South Asian states of Bangladesh, India, and Pakistan [14,15]. Most establishmentarians have evaluated that a minimum 5.2% of the global populace (over 300-360 million) bear structurally irregular hemoglobin [14,16]. There are almost 80-90 million carriers of β-thalassemia globally, accounting for 1.5% of the world population. Additionally, it has been reported that around 68,000 infants are born yearly with β-thalassemia, both minor and major [17]. The population with thalassemia carrier status varies from country to country. It is approximately 6-12% of the population in Bangladesh [14], approximately 6.8-12.8% of the population in Malaysia [12,18], and about 40% of the population in Thailand [19]. Multiple studies reported that around 23,000 as thalassemia major and 90% of these children are born in low or middle-income countries (LMICs) [3,6].

Complications of thalassemia and related issues

**Figure 2:**
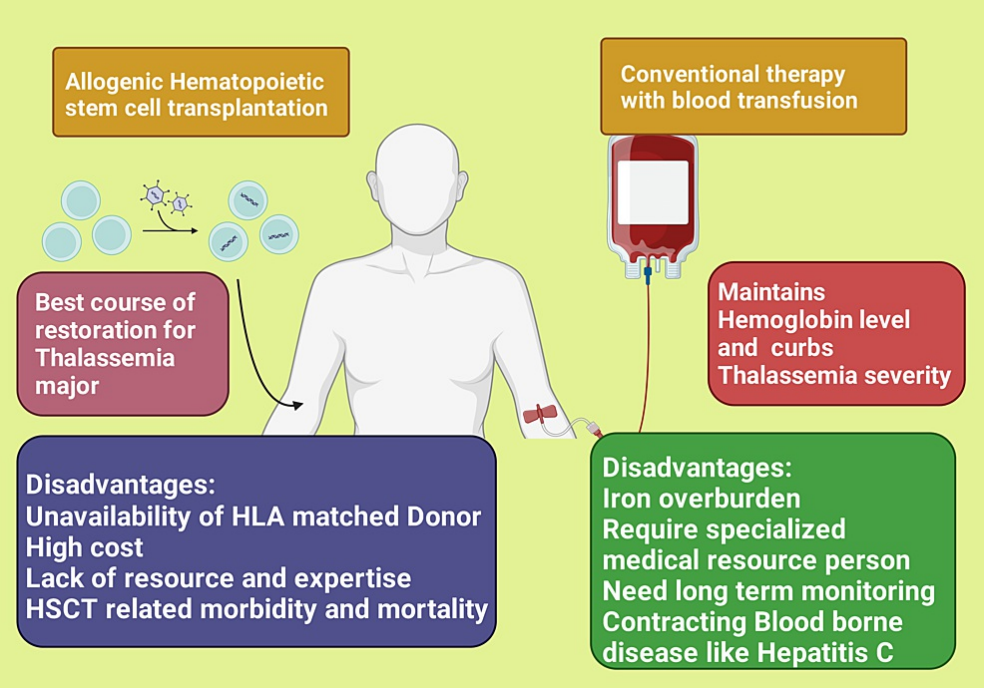
Showing the advantage and disadvantages of the different treatment options for thalassemia HLA: human leukocyte antigens; HSCT: hematopoietic stem cell transplantation This figure has been created using the premium version of Biorender (https://biorender.com/) with publication license no.: BL24OA622P. Image Credit: Rahnuma Ahmad

The burden of thalassemia on the patients is due to the complications from the disease itself or the consequence of treatment received by the patient. Transfusion-dependent thalassemia patients ultimately cause iron overburden which calls for iron chelation therapeutic interference. Long-term monitoring and management should be conducted by medical resource persons, especially hematologists [20]. Allogenic hematopoietic stem cell transplantation (HSCT) offers the best restorative course of action for major thalassemia cases. However, it is mostly not feasible for the majority of thalassaemic cases on account of impediments or the unavailability of HLA-matched donors, lack of resources and expertise, high cost, and the high risk of HSCT-related mortality and morbidity [1,21,22] (Figure 2). Nowadays, there is a considerable advancement in the treatment of thalassemia patients in terms of blood transfusions and iron chelation therapy resulting in a declining mortality rate in Western countries from 12.7 deaths/1000 patients during the years 1980-1999 to 1.65 deaths/1000 patients during the years 1999-2013. However, there still are high morbidities with advanced age due to exposure to disease and treatment-related side effects such as complications of transfusion and transplantation, hepatitis C exposure, complications related to iron overload, bone disease, endocrine disease, and other complications, and premature death [23,24]. Transfusion-dependent patients were shown to have a poor health-related quality of life compared to non-thalassemia patients due to underlying complications such as splenectomy, diminutive build, malnourishment, and prolonged hospitalization [21]. They were reported to express hopelessness, low self-esteem, low intelligence quotient, poor school performance, and social restriction [25]. Patients and their family members, especially mothers, suffer intense physical, mental, and social trauma [25,26].

## Review

In this review, we have highlighted the challenges experienced by the parents and caregivers. The suffering of children due to this chronic disease imposes a great social, psychological, and financial burden on the parents and families [27]. The family feels the burden owing to the long-standing character of the sickness, the treatment modalities, the complications and mortalities caused by the disease, and the need for frequent visits to healthcare centers for blood transfusions and other necessary blood investigations [28]. Parents reported that the life-long treatment modality of blood transfusion and iron chelation therapy, especially the complications following transfusion, make the parents and caregivers psychologically distressed and frustrated. The complication of the disease itself, causing physical changes impacting the children's day-to-day activities, adds more distress to the parents. This distress starts with diagnosing the disease in children [19,29]. 

**Figure 3:**
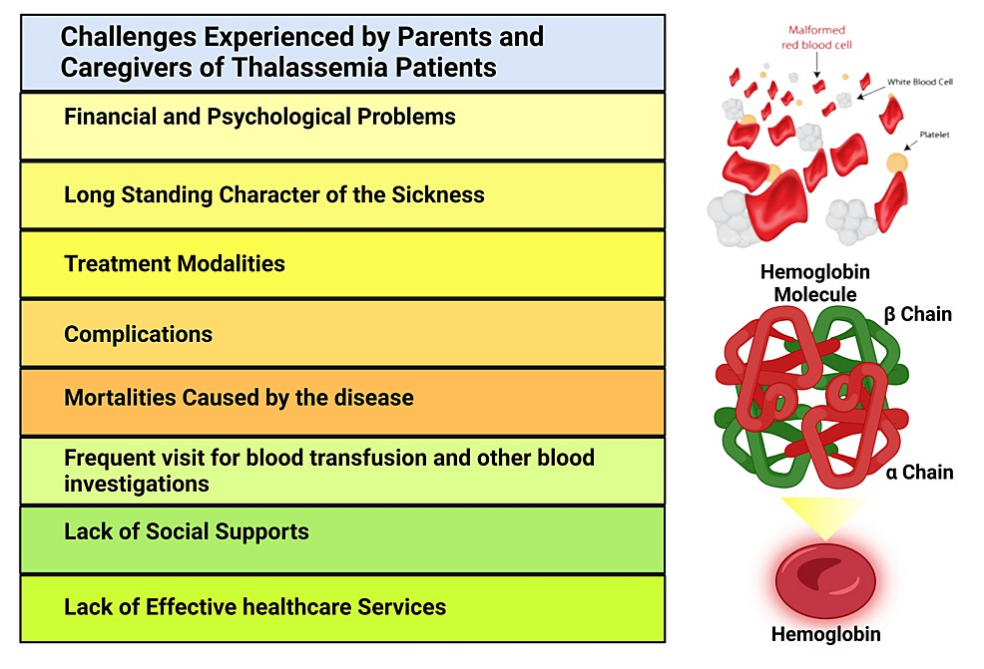
Different Problems Endured by Parents and Caregivers of Thalassemia Patients. This figure has been developed using the premium version of Biorender (https://biorender.com/) with the publication license number: BH24O9UVHQ. Image Credit: Susmita Sinha

A study conducted in Thailand showed mothers were burdened due to the defeatist influence of this persistent disease on the quality of life. The study showed that mothers encounter financial and psychosocial problems, lack of social support, lack of effective healthcare services, and worry about their children's future. This serious chronic disease negatively impacts their children's school and studies, causes limitations in playing with friends, and causes complications and discomfort from blood transfusion (Figure 3) [19]. Multiple studies conducted in the Middle East reported that parents suffer mentally and physically. They feel guilty about the disease, suffer from insomnia and fatigue; they are concerned about the future of their children, and often conceal the disease due to societal faith that it has negative bearings on the family honor, and have a low level of awareness and know-how regarding thalassemia in society, as well as feeling a financial burden [27,28]. Parents feel that they become socially victimized and stigmatized as they feel that they lack a support system, and experience constant stress which requires coping strategies. All these issues ultimately cause immense suffering to the parents and caregivers [30]. The important issue of social death and stigmatization was found to be more prevalent in Iran. There are false beliefs and superstitions about the disease due to cultural beliefs, stigma, and lack of knowledge about the disease [27,30]. The social attitude, shame, and stigmatization towards the patient cause the parents to conceal the disease and secretly pursue the treatment and care of their children. The parents and the family become socially isolated and communicate poorly [27]. The other reason that causes parents to become socially isolated and contributes to the impairment of social relations is that they need to spend more time constantly with their children [28,29]. They are often not able to fulfill their job requirements and other daily activities. This imposes immense pressure mentally, financially, and physically on them [31]. Another similar study in Athens showed mothers of children with thalassemia were concerned about psychological agony and fear of death, and had trouble managing emotions, although they were reported to have received support [32]. Social support to the patient and parents is important in managing thalassemia. Showing respect to the individual and kind words from the neighbors, friends, and community are the basis for this support [19]. Lack of social support causes immense psychosocial burdens to patients and families. Therefore, support from family, friends, community, and medical professionals is essential to confront the disease and overcome the burden [33].

In thalassemia major patients, the financial burden is prominent, like any chronic disease, due to follow-up visits, treatment, and frequent admissions, besides the transportation cost and high living cost [19,29,34]. This is more problematic for parents in low socio-economic countries with families having poor financial conditions with limited income. As the parents often bear the cost for the entirety of their child's life, it puts them under a tremendous financial burden [35]. Moreover, parents are busy with their children’s care, and their scope of income becomes more restricted. Increased expenses due to the treatment and other related costs add more to this financial difficulty [19]. Fathers especially bear this burden as in many families, due to cultural norms, it is the responsibility of the father to bear all the expenses, including living costs [34].

Treatment cost of thalassemia

It is reported that the cost of transfusion in a nonsubsidized hospital is higher than the average family income, which gives rise to a financial load both on the patients, their families, and their communities [14]. The cost of treatment in different countries was studied. In Sri Lanka, the average annual cost to manage thalassemia patients is approximately US $2601 per patient-year [36]; in Iran, it is about US $8321 per patient [37], while in Thailand, it is US $950 per patient [38]. In India, it ranged from US $108-432 per patient-year [39], while in Bangladesh the cost of blood transfusion is only about US $64.72 to US $411.17 per year [40]. This data explains the financial strain borne by the family. There are some countries that provide financial support [19, 27]. However, this support is lacking in other countries. These countries need to focus more on reducing the financial burden by providing financial support and protection. Support from national healthcare providers is necessary as well, and charitable organizations and nongovernmental organizations are required to come forward to overcome this financial burden [27]. Financial support by exempting hospital bills and some support from the government would be helpful [34]. 

Stress related to the treatment of thalassemia

Parents worrying about their children's future is a major finding of many studies as well as concerns about the children's future health status. They want their children to be strong and healthy. They were concerned especially about the employment of their children. Even after employment, the children’s ability to do heavy work is also a great concern for the parents [19,27,34]. Parents also feel uncertain about their children's future in aspects like education, marriage, and having children [34]. 

It is found that mothers suffered physical and psychological stress and ignored their own diseases and did not take appropriate measures due to lack of time [41]. A study among Jordanian mothers having children with thalassemia revealed that mothers face various forms of stress, such as non-physical and cognitive stress, social segregation, and concerns about their children's future, which are further aggravated due to deficiency of knowledge and financial burden [29]. A study conducted in Iraq showed that caregivers who are mainly mothers (55%) feel afraid of the complications of the disease, feel guilty, and are frustrated, causing a loss of pleasure in life [28]. Mothers suffer from low-level quality of life with low physical and mental health and a sense of guilt due to the inheritance of this genetic disease [25]. It is necessary to provide support to these mothers by relatives, family members, community people, and also the health care providers by providing counseling to enhance their self-care knowledge [41].

Educational and psychological support with counseling

Although the current management of thalassemia has improved substantially, it is now vital to give due importance to improving the psychosocial problems faced by the patient, caregivers, and family members [28]. Lack of knowledge of the causes of disease and its treatment, along with social, cultural, and religious factors, adds to the psychosocial burden. Therefore, the efficacious therapeutic intervention of thalassemia requires an all-inclusive scientific understanding, interpretation, and perception regarding β-thalassemia major [33]. Long-term psychosocial support is required to help reduce emotional distress, improve compliance with treatment and strengthen coping strategies to improve the quality of life for thalassaemic cases and related family members [25,34]. Mothers and caregivers must be supported through appropriate counseling programs and enhancing self-care knowledge. This can be done by psychologists and psychiatric nurses in identifying, helping, and supporting mothers of pediatric thalassaemic cases with emotional issues [41]. Holistic nursing interventions can support parents and motivate them to cope with their psychosocial and physical burdens. A good example of such intervention is by arranging programs to educate parents about taking care of their children [30]. Thus, disseminating knowledge regarding the disease by healthcare professionals through regular educational programs would help mothers and caregivers prevent or reduce their psychosocial problems [19]. A digital thalassemia database with all the necessary information regarding thalassemia will be of great benefit. This will allow the parents to immediately access information such as disease processes or therapeutic interventions and preventions [42].

**Figure 4:**
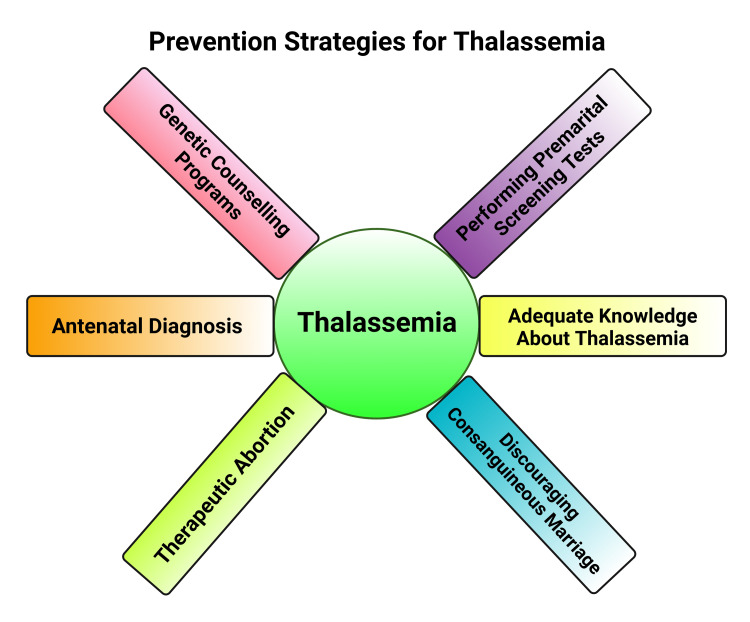
Prevention Strategies for Thalassemia. This figure has been developed using the premium version of Biorender (https://biorender.com/) with the publication license number: CE24O8MGQB. Image Credit: Susmita Sinha

More emphasis must be given to prevention by performing screening tests and antenatal diagnosis [11]. One of the important strategies of the health care system is genetic counseling which helps to decrease the frequency of new patients. Therefore, genetic counseling programs play a great role in preventing the disease. Genetic counseling allows the parents to have a better understanding of the nature of the disease, its consequences, transmission, risks, and ways of prevention of transmission, and thus the parents can take the right decision regarding the ways of prevention, such as adopting family planning, antenatal diagnosis or performing a therapeutic abortion, etc. Genetic counseling intervention helps parents to ease their suffering and solve their problems [30]. Lack of knowledge, ignorance of the disease, and lack of premarital screening practices play a major role in the propagation of this disease [43]. Thalassemia carriers are mostly unaware of the disease condition that actually acts as a source of disease. Only premarital screening can detect this condition [43]. However, many people are reluctant or afraid to do the screening test due to societal norms and its adverse effect on the marriage prospectus or due to ignorance about the disease [43]. Consanguineous marriage among first cousins has a significant role in the continuation of the disease process. Studies have shown that most parents with thalassemic children were found to have consanguineous marriages [29,33,43]. Therefore, it is required to emphasize developing awareness of the disease and encourage carrier detection by doing premarital screening and counseling to reduce the disease frequency among the population (Figure 4). Although the pathology of thalassemia possesses diverse molecular variance; nevertheless, it is currently attainable to achieve precise diagnosis with efficient DNA-based techniques, especially for β-thalassemia [44]. There have been salient breakthroughs regarding gene therapeutic intervention for β-thalassemia [45].

Hematopoietic stem cell transplantation and gene therapy

Multiple studies reported that single-gene disorders β-thalassemia and sickle cell anemia (SCA) have the possibility to be cured through allogeneic hematopoietic stem cell transplantation (HSCT) [46-48]. Gene therapy with autologous CD34+ cells (transmembrane phosphoglycoprotein protein encoded by the CD34 gene in humans) transduced with the BB305 vector (an exploratory gene therapeutic agent for the management of sickle cell disease and β-thalassemia) diminishes or puts an end to the necessity for long-standing red-cell transfusion therapy among transfusion-dependent β-thalassemia patients [49,50].

## Conclusions

Thalassemia causes immense psychosocial, physical, and financial burdens to patients and their families. It is required to enhance awareness by increasing the knowledge of the parents and caregivers about thalassemia. Educational programs are required to enhance mothers’ awareness about self-care and taking care of their ill children. Awareness among close family members, relatives, friends, and the community would also be extremely helpful for social support. Financial support is required to lessen their stress. A multidisciplinary approach to managing the disease and psychosocial support is required for the management of thalassemia and to minimize the burdens on both patients and families. Prevention by making premarital screening and carrier identification through preimplantation, and genetic diagnosis (PGD) for β-thalassemia with or without HLA typing as well as genetic counseling for the parents to take appropriate measures will help to lessen the propagation of this disease.
